# The Predictive Prognostic Values of Serum TNF-**α** in Comparison to SOFA Score Monitoring in Critically Ill Patients

**DOI:** 10.1155/2013/258029

**Published:** 2013-09-19

**Authors:** Ayman Abd Al-Maksoud Yousef, Ghada Abdulmomen Suliman

**Affiliations:** Faculty of Medicine, Tanta University, 17 Elemam Moslem Street, Tanta 35217, Egypt

## Abstract

*Background*. The use of inflammatory markers to follow up critically ill patients is controversial. The short time frame, the need for frequent and serial measurement of biomarkers, the presence of soluble receptor and their relatively high cost are the major drawbacks. Our study's objective is to compare the prognostic values of serum TNF-**α** and SOFA score monitoring in critically ill patients. *Patients and Methods*. A total of ninety patients were included in the study. Forty-five patients developed septic complication (sepsis group). Forty-five patients were critically ill without evidence of infectious organism (SIRS group). Patients' data include clinical status, central venous pressure, and laboratory analysis were measured. A serum level of TNF-**α** and SOFA score were monitored. *Results*. Monitoring of TNF-**α** revealed significant elevation of TNF-**α** at 3rd and 5th days of ICU admission in both groups. Monitoring of SOFA score revealed significant elevation of SOFA scores in both groups throughout their ICU stay, particularly in nonsurvivors. Positive predictive ability of SOFA score was demonstrated in critically ill patients. *Conclusion*. Transient significant increase in serum levels of TNF-**α** were detected in septic patients. Persistent elevation of SOFA score was detected in nonsurvivor septic patients. SOFA score is an independent prognostic value in critically ill patients.

## 1. Introduction

Despite the advances in management of critically ill patients, sepsis remains one of the leading causes of deaths among ICU population representing about 50% of total ICU mortality. Care of septic patients represents a great economic burden as extraordinary resources are directed towards new potential treatment besides novel diagnostic and prognostic tools [1].

Prediction of ICU morbidity and mortality is a very challenging process. Outcome prediction could provide useful information regarding therapeutic decision making and guide resource allocation [2]. 

Soluble tumor necrosis factor-*α* (TNF-*α*) had been established as an important crucial cytokine in inflammatory states including sepsis and SIRS, but its frequent monitoring is helpful to reveal the onset and to predict the outcome of septic patients. However, liability of serum dynamics of TNF-*α* reflects debate about its ability to evaluate changes in patients' status over time [3, 4]. 

The sequential organ failure assessment (SOFA) score allows for calculation of organ dysfunction of six organ systems, in addition to the severity of these dysfunctions. These organs are respiratory, coagulation, liver, cardiovascular, renal, and neurologic systems. SOFA score could be useful in providing therapeutic decision making and guiding resource allocation [5]. 

The present study was conducted to determine the dynamic changes of serum TNF-*α* and the corresponding changes of serial evaluation of SOFA score and their correlation in critically ill sepsis and SIRS patients. 

## 2. Materials and Methods

After the study was approved by the Institutional Review Board of Faculty of Medicine, Tanta University, an informed consent was obtained from patients participating in the study or their relatives. The study was conducted in the ICU of the Emergency Hospital of Tanta University, Tanta, Egypt. A total of ninety patients (52 men and 38 women) were included in the study. Forty-five patients developed septic complication during ICU stay (sepsis group). Forty-five patients were critically ill without evidence of infectious organism (SIRS group). Patients were classified into their groups at the time of the first blood analysis for their biomarkers at ICU admission. 

The patients staying in ICU for more than 24 hours were enrolled in the study. Patients received anti-inflammatory drugs or corticosteroids before admission, patients had immunosuppressive illness, patients had chronic organ failure, patients received massive blood transfusion, patients with radiation therapy and patients with previous organ transplantation were excluded from the study. At admission, patients' age, sex, weight, and height were recorded. Patients' data that include the clinical status, sequential organ failure assessment (SOFA) score, temperature, heart rate, respiratory rate, blood pressure, central venous pressure, laboratory analysis (complete blood count, blood urea nitrogen, blood sugar, serum sodium, potassium, calcium, aspartate aminotransferase, alanine aminotransferase, prothrombin time, albumin, and CRP), and arterial blood gas analysis were measured. Serum TNF-*α* was monitored at admission, 3rd, 5th and 7th day of ICU stay. In addition, routine cultures of suspected sites, blood, and urine were obtained at admission and whenever necessary to determine the presence of infection. We attempted to maintain the patient hemoglobin level at 10–12 g/dL and central venous pressure at 8–12 cm H_2_O. When needed, intravascular fluid replacement, blood products, and inotropic or vasopressor agents were administered. 

Each day the attending physician evaluated all the study patients for sepsis, severe sepsis, or septic shock throughout their stay in ICU. The signs of sepsis were body temperature <36C° or >38C°, tachycardia (>90 beats/min), ventilatory frequency >20 breath/min or PaCO_2_ <32 mmHg (unless the patient was mechanically ventilated), a white cell count ≥12 × 10^9^ litre^−1^ or <4 × 10^9^ litre^−1^, or >10% immature neutrophils, in addition to the presence of infection. Severe sepsis is sepsis associated with evidence of organ dysfunction, hypoperfusion, acute alteration of mental status, elevated plasma lactate, unexplained metabolic acidosis (arterial ph < 7.3), hypoxaemia, prolonged prothrombin time or decrease in platelet count >50% or ≤100 × 10^9^ litre^1^, oliguria, and hypotension defined as systolic arterial pressure <90 mmHg or a decrease of >40 mmHg. Septic shock was defined as hypotension (<90/60 mmHg) in addition to sepsis syndrome persisting despite adequate fluid resuscitation and inotropic support [6].

### 2.1. Blood Sampling

Blood samples were collected in glass tubes. Blood was processed within two hours. It was centrifuged at 1,600 g for 15 minutes.

#### 2.1.1. TNF-***α*** Determination Using ELISA

A serum level of TNF-*α* was determined by quantitative sandwich enzyme immunoassay (R&D Systems, Inc., Minneapolis, MN, USA) guided by the manufacturer's instructions. The intensity of the color was measured at 490 nm.

#### 2.1.2. Evaluation of SOFA Score

 SOFA score is composed of scores of six organ systems (respiratory (R), cardiovascular (C), hepatic (H), coagulation (Co), renal (Re), and neurological (N)) graded from 0 to 4 according to the degree of dysfunction/failure [6] (Table 1).

### 2.2. Statistical Analysis

Parametric data were analyzed using either ANOVA or Student's *t*-test while nonparametric data were analyzed using Mann-Whitney *U* and *χ*_2_-tests. Data were presented as mean and standard deviation. A *P* value of <0.05 was considered significant.

### 2.3. Sample Size Analysis

We calculated that we need 43 patients per group to have an 80% chance of detecting a 25% change in serum TNF-*α* at a 5% significance level with a 2-sided significance level (nQuery Advisor, Version 5.0), so we included 45 patients per group.

## 3. Results

A total of ninety patients (52 men and 38 women) were included in the study. Forty-five patients developed septic complication during ICU stay (sepsis group). In this group, seven patients developed septic shock, ten developed severe sepsis, and twenty-eight patients developed sepsis without any organ dysfunction. Forty-five patients were critically ill without evidence of infectious organism (SIRS group). Twenty-one patients died, seven of them were in septic shock, ten were suffering from severe sepsis, and four cases died in SIRS group. There was no significant difference between groups, except for the duration of the stay in the ICU which was higher in septic patients (Table 2). 

 Serum monitoring of TNF-*α* in sepsis group revealed a significant elevation on the 3rd and 5th days of ICU admission, the initial mean value was 75.7 ± 15.1 pg/mL, the 3rd day mean value was significantly elevated to 311.7 ± 133 pg/mL (*P* = 0.001), the 5th day mean value was 237.7 ± 101 pg/mL (*P* = 0.003), while the 7th day mean value was 116.88 ± 44 pg/mL (*P* = 0.85). 

Serum monitoring of TNF-*α* in SIRS group revealed the significant elevation on the 3rd and 5th days of ICU admission, the initial mean value was 72.44 ± 18 pg/mL, 3rd day mean value was significantly elevated to 153.8 ± 52.3 pg/mL (*P* = 0.025), 5th day mean value was 122.2 ± 28.1 pg/mL (*P* = 0.034), while 7th day mean value was 79.3 ± 14.5 (*P* = 0.72) (Table 3). 

In addition, the mean value of TNF-*α* at admission in sepsis group is statistically insignificant in comparison to SIRS group (*P* = 0.15). The 3rd and 5th days of ICU stay mean values were significantly higher in sepsis group (*P* = 0.007 and 0.022, resp.), while 7th day mean value was statistically insignificant between both groups (*P* = 0.179) (Table 3).

Monitoring of SOFA score in sepsis group revealed the significant elevation on the 3rd, 5th, and 7th days of ICU admission: the initial mean value was 5.9 ± 1.43, the 3rd day mean value was significantly elevated to 8.26 ± 1.57 (*P* = 0.03), the 5th day mean value was 10.9 ± 2.7 (*P* = 0.025), and the 7th day mean value was 11.31 ± 4.74 (*P* = 0.015) (Table 4). 

Monitoring of SOFA score in SIRS group revealed nonsignificant elevation during ICU stay, the initial mean value was 4.35 ± 1.19, 3rd day mean value was 5.37 ± 1.07, 5th day mean value was 6.06 ± 1.6, while the 7th day mean value was 4.57 ± 1.9 (*P* = 0.41, 0.36 and 0.45) (Table 4).

 In addition, the mean value of SOFA score at admission, 3rd, 5th, and 7th days of ICU stay was significantly higher in sepsis groups when compared to mean values in SIRS group (*P* = 0.036, 0.028, 0.019, and 0.005, resp.) (Table 4). 

 Concerning survival rate for sepsis and SIRS groups, twenty-eight patients are survivors in sepsis group, while seventeen patients are nonsurvivors during their ICU stay. Regarding SIRS group, forty one are survivors while four are nonsurvivors during their ICU stay. There was a significantly higher number of nonsurvivors in sepsis group (*P* = 0.001) (Table 5). 

In sepsis group, the admission mean values of TNF-*α* in survivors revealed nonsignificant change to admission mean value in nonsurvivors (*P* = 0.435). The 3rd and 5th day mean values were significantly higher in nonsurvivors (*P* = 0.035 and 0.039, resp.). The 7th day mean values revealed nonsignificant change between survivors and nonsurvivors (*P* = 0.063) (Table 6).

In SIRS group, the admission mean values of TNF-*α* in survivors revealed nonsignificant change to admission mean value in nonsurvivors (*P* = 0.103). The 3rd and 5th day mean values were significantly higher in nonsurvivors in comparison to survivors (*P* = 0.025 and 0.034, resp.), while the 7th day mean value revealed nonsignificant change (*P* = 0.58) (Table 6). 

The 5th and 7th day mean values of SOFA score were significantly higher in nonsurvivors in comparison to survivors in sepsis group (*P* = 0.013 and 0.004) (Table 7). 

The 5th and 7th day mean values were significantly higher in nonsurvivors in comparison to survivors in SIRS group (*P* = 0.032 and 0.015) (Table 7). 

Logistic regression analysis revealed that the peak value of SOFA score has significant positive prediction of mortality (*P* = 0.006), while the peak value of TNF-*α* is not significantly correlated with mortality (Table 8). 

**Figure 1:**
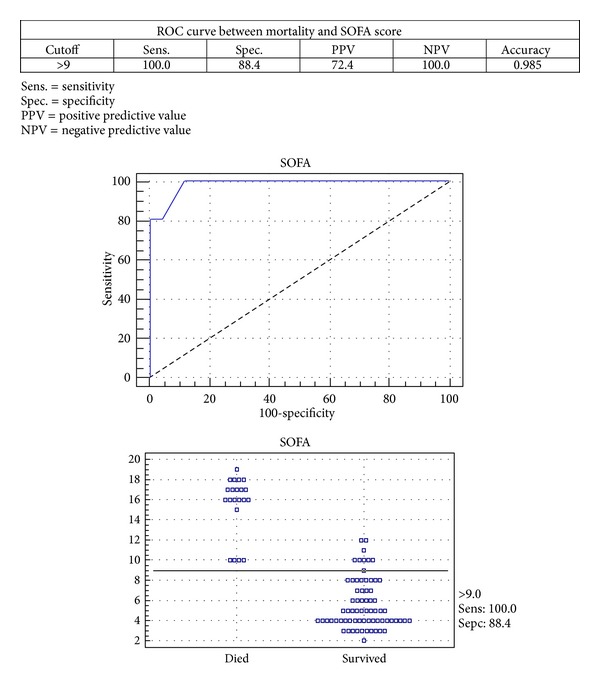
Receiver operator curve for correlation of mortality and SOFA score.

The receiver operator curve for SOFA score regarding mortality revealed that SOFA score cutoff value is 9, sensitivity of 100%, specificity of 88.4%, and accuracy of about 0.985 (Figure 1).

## 4. Discussion

This study is the first to evaluate simultaneously both the serum TNF-*α* dynamics in critically ill patients in addition to the corresponding changes in SOFA score in critically ill patients which indirectly reflect the clinical severity status of the patients and the degree of organ systems dysfunction. A transient significant elevation of TNF-*α* was observed in sepsis and SIRS patients during the 3rd and 5th days of ICU stay in relation to their admission mean values. TNF-*α* showed transient significant elevation in nonsurvivor sepsis and SIRS patients in comparison to survivors. While SOFA score is predominately significantly higher in septic patients throughout their ICU stay in comparison to SIRS patients, the SOFA score is significantly elevated in non-survivors sepsis and SIRS patients in comparison to survivors. A positive correlation between the peak value of TNF-*α* during their ICU stay correlates with their peak SOFA score mean values. 

Accurate prognostic indicators for patients' survival in ICU are important and helpful to guide clinical decision making. The development of several scoring systems enabled the critical care physician to accurately and reliably measure the severity of illness in ICU. The scoring system in ICU should assess various degrees of organ dysfunction starting from normal function to organ failure; assessment of organ dysfunction needs to be based on simple, easy repeatable variables specific to the desired organ, readily available and able to reflect the dynamic changes of illness over time [2]. SOFA score has been used to determine individual severity; it allows for repeated measurements of multiple organ dysfunction or failure and consequently, acts as an index for determining either sequential improvement or deterioration of the pathological status of the patients during their ICU stay [7]. 

The elevation of TNF-*α* production does not seem to correlate with severity of clinical status in both sepsis and SIRS critically ill patients, making the elevation of TNF-*α* production seems not to be an independent predictor in patients with sepsis. Thus, the biological function of TNF-*α* is largely influenced by two TNF-*α* receptors that are soluble TNF receptors (sTNFR) and cell surface TNF receptors (cTNFR) [8].

Previous studies demonstrated that SOFA score assessment during ICU stay is a good indicator of prognosis in ICU critically ill patients [4]. Jones et al. [9] concluded in their study that SOFA score provides potentially valuable prognostic information on in-hospital survival when applied to patients with severe sepsis. However, both studies did not correlate the SOFA score with other inflammatory markers. Presterl et al. [10] demonstrated a correlation between the plasma level of CRP, IL-6, TNF, APACHE III and mortality probability models II scores. Both scoring systems as well as CRP were significantly higher in nonsurvivors compared with survivors. Zygun et al. [11] demonstrated that SOFA score was higher in nonsurvivors than survivors at the time of ventilator associated pneumonia. Gursel and Demirtas [12] concluded that in patients with brain injury, the SOFA scoring system has superior discriminative ability and stronger association with outcome with respect to hospital mortality and unfavorable neurologic outcome. Minne et al. [13] advocated the use of combination of a traditional model based on data from the first 24 hours after ICU admission, like APACHE IV with SOFA scores to improve prediction of mortality. Cholongitas et al. [14] in their study concluded that SOFA score had better predictive ability in cirrhotic patients admitted to intensive care unit. Ceriani et al. [15] concluded that SOFA score may be used to grade the severity of postoperative morbidity in cardiac surgical patients, the model identifies patients at increased risk for postoperative mortality. Serial measurement of SOFA score in critically ill patients may help to identify patients who may require more aggressive therapeutic intervention and to avoid complications.

## 5. Conclusion

Transient significant increase in serum levels of TNF-*α* was detected in nonsurvivors septic patients. Persistent elevation of SOFA score was detected in nonsurvivor septic patients. The peak value of SOFA score is an independent prognostic value in critically ill patients.

## Figures and Tables

**Table 1:** SOFA score component.

Variables/scores	0	1	2	3	4
Respiratory (paO_2_/FIO_2_, mmHg)	≥400	≤400	≤300	≤200	≤100
Coagulation (PlT ×10^3^/*µ*L)	≥150	≤150	≤100	≤50	≤20
Liver (bilirubin, mg/dL)	≥1.2	1.2–1.9	2–5.9	6–11.9	≤12
CNS GCS	15	13–14	10–12	6–9	≥6
Renal (creatinine, mg/dl)	≥1.2	1.2–1.9	2–3.4	3.5–4.9	≤5
Cardiovascular	MAP ≥ 70 mmHg	MAP ≤ 70 mmHg	Dop ≤ 5 Mic/kg/min	Dop ≥ 5 Epi ≤ 0.1 Mic/kg/min	Epi ≥ 0.1 Mic/kg/min

MAP: mean arterial pressure, DOP: dopamine, Epi: epinephrine, CNS: central nervous system.

**Table 2:** Patient characteristics (mean and standard deviation).

	Sepsis group (*n* = 45)	SIRS group (*n* = 45)
Age (years)	62.8 ± 14.5	59.6 ± 12.6
Sex ratio (M/F)	25/20	27/18
Duration of ICU stay (day)	15.5 ± 4.7*	4.2 ± 1.9
Diagnosis		
Respiratory insufficiency	16	15
Polytrauma	14	15
Orthopedic surgery	9	10
Thoracic surgery	6	5

*Significant change (*P* < 0.05).

**Table 3:** Comparison of tumor necrosis factor-*α* in the studied groups.

	SEPSIS	SIRS
	Mean ± SD	Mean ± SD
TNF-*α* 1	75.778 ± 15.112	72.444 ± 18.079
TNF-*α* 3	311.711^∗†^ ± 133.048	153.889* ± 52.374
TNF-*α* 5	237.756^∗†^ ± 101.108	122.222* ± 28.175
TNF-*α* 7	116.889 ± 44.445	79.333 ± 14.523

*Statistically significant within group (*P* < 0.05).

^†^Statistically significant between groups (*P* < 0.05).

**Table 4:** Comparison of  SOFA score in the studied groups.

	SEPSIS	SIRS
	Mean ± SD	Mean ± SD
SOFA-1	5.933^†^ ± 1.437	4.356 ± 1.190
SOFA-3	8.267^∗†^ ± 1.572	5.378 ± 1.072
SOFA-5	10.9^∗†^ ± 2.796	6.067 ± 1.643
SOFA-7	11.311^∗†^ ± 4.743	4.578 ± 1.948

*Statistically significant within group (*P* < 0.05).

^†^Statistically significant between groups (*P* < 0.05).

**Table 5:** Comparison of survival in the studied groups.

		SEPSIS	SIRS	Total
Survived	*N*	28	41	69
	%	62.22	91.11	76.67

Nonsurvived	*N*	17	4	21
	%	37.78	8.89	23.33

Total	*N*	45	45	90
	%	100.00	100.00	100.00

Chi-square	*χ* ^2^	11.126		
	*P* value	0.001		

**Table 6:** Comparison of serum TNF-α in survived and nonsurvived patients in the studied groups.

	Groups	Survived	Nonsurvived
		Mean ± SD	Mean ± SD
TNF-*α* 1	SEPSIS	72.321 ± 16.470	81.471 ± 10.719
	SIRS	72.439 ± 18.779	72.500 ± 9.574

TNF-*α* 3	SEPSIS	238.643^∗†^ ± 74.700	432.059* ± 120.546
	SIRS	142.683^∗†^ ± 39.324	268.750* ± 17.500

TNF-*α* 5	SEPSIS	179.107^∗†^ ± 54.230	334.353* ± 84.950
	SIRS	116.585^∗†^ ± 20.780	180.000* ± 31.623

TNF-*α* 7	SEPSIS	95.893 ± 27.990	110.471 ± 45.475
	SIRS	77.927 ± 14.317	93.750 ± 7.500

*Statistically significant within the same group (*P* < 0.05).

^†^Statistically significant between both groups (*P* < 0.05).

**Table 7:** Comparison of SOFA score in survived and nonsurvived patients in the studied groups.

	Groups	Survived	Nonsurvived
		Mean ± SD	Mean ± SD
SOFA-1	SEPSIS	5.714 ± 1.536	6.294 ± 1.213
	SIRS	4.317 ± 1.128	4.750 ± 1.893

SOFA-3	SEPSIS	7.821 ± 1.679	9* ± 1.061
	SIRS	5.244 ± 1.019	7.750 ± 0.500

SOFA-5	SEPSIS	8.464 ± 2.063	12.529^∗†^ ± 1.841
	SIRS	5.756 ± 1.338	9.250^∗†^± 0.957

SOFA-7	SEPSIS	7.929 ± 2.142	16.882^∗†^ ± 1.054
	SIRS	4.049 ± 0.973	10.000^∗†^ ± 0.855

*Statistically significant within group (*P* < 0.05).

^†^Statistically significant between groups (*P* < 0.05).

**Table 8:** Logistic regression analysis for mortality prediction of peak values of TNF-*α* and SOFA score in both groups.

Logistic regression	*B*	S.E.	Wald	*P* value	Odd	95.0% C.I. for odd	
						Lower	Upper
TNF-*α* 3	0.002	0.009	0.071	0.790	1.002	0.985	1.020
SOFA-7	0.839	0.304	7.628	0.006	2.315	1.276	4.200
Constant	−10.358	3.012	11.827	0.001	0.000		
